# Ablation of Bax and Bak protects skeletal muscle against pressure-induced injury

**DOI:** 10.1038/s41598-018-21853-5

**Published:** 2018-02-27

**Authors:** Bjorn T. Tam, Angus P. Yu, Eric W. Tam, Douglas A. Monks, Xu P. Wang, Xiao M. Pei, Su P. Koh, Thomas K. Sin, Helen K. W. Law, Felix N. Ugwu, Rashmi Supriya, Benjamin Y. Yung, Shea P. Yip, S. C. Wong, Lawrence W. Chan, Christopher W. Lai, Pin Ouyang, Parco M. Siu

**Affiliations:** 1 Department of Health Technology and Informatics, Faculty of Health and Social Sciences, The Hong Kong Polytechnic University, Hong Kong, China; 2 School of Public Health, Li Ka Shing Faculty of Medicine, The University of Hong Kong, Hong Kong, China; 3 Interdisciplinary Division of Biomedical Engineering, Faculty of Engineering, The Hong Kong Polytechnic University, Hong Kong, China; 4 Department of Cell and Systems Biology & Department of Psychology, University of Toronto, Toronto, Canada; 5 The Key Laboratory of Cardiovascular Remodelling and Function Research, Chinese Ministry of Education and Chinese Ministry of Health, Qilu Hospital, Shandong University, Jinan, Shandong China; 6 Department of Integrative Biology and Pharmacology, The University of Texas Health Science Center at Houston, Houston, Texas USA; 7 Department of Anatomy & Transgenic Mouse Core Laboratory, Chang Gung University, Taoyuan, Taiwan

## Abstract

Pressure-induced injury (PI), such as a pressure ulcer, in patients with limited mobility is a healthcare issue worldwide. PI is an injury to skin and its underlying tissue such as skeletal muscle. Muscle compression, composed of mechanical deformation of muscle and external load, leads to localized ischemia and subsequent unloading reperfusion and, hence, a pressure ulcer in bed-bound patients. Although the gross factors involved in PI have been identified, little is known about the exact disease mechanism or its links to apoptosis, autophagy and inflammation. Here, we report that PI is mediated by intrinsic apoptosis and exacerbated by autophagy. Conditional ablation of Bax and Bak activates the Akt-mTOR pathway and Bnip3-mediated mitophagy and preserves mitochondrial contents in compressed muscle. Moreover, we find that the presence/absence of Bax and Bak alters the roles and functions of autophagy in PI. Our results suggest that manipulating apoptosis and autophagy are potential therapeutic targets for treatment and prevention of PI.

## Introduction

Mechanical deformation of muscle by external load leads to localized ischemia and subsequent unloading reperfusion and, hence, a pressure ulcer^1^. Although the gross factors involved in PIs have been identified^2,3^, the exact disease mechanisms and therapeutic targets remain unknown. We have previously reported that apoptosis is involved in PI pathogenesis and that pharmacological inhibition of caspases using the pan-caspase inhibitor z-VAD-fmk alleviates muscle injury induced by mechanical compression^4,5^, suggesting that pressure ulcers are probably mediated by apoptosis. Besides apoptosis, autophagy is another stress response pathway that is likely to be activated in PIs. It has been generally accepted that autophagy, a highly conservative intracellular mechanism recycling organelles and long-lived proteins, and apoptosis function simultaneously in the cell although the stress-induced autophagy comes before apoptosis in most of the cases^6^. It implies that autophagy is activated to cope with the stresses, including mechanical, chemical and radiation insults. However, when the stress is not removed or alleviated and no longer tolerable, apoptosis could be activated. At the same time, the highly activated autophagy may have already depleted a significant portion of cellular content, leading to autophagic cell death^7^. It seems that the roles of autophagy in the stressed cells vary depending on the dose, intensity and duration of stress^8^. Apart from the pro-death and pro-survival functions of autophagy, whether Bcl-2 inhibits autophagy directly by interacting with Beclin-1 or indirectly by inhibiting Bax and Bak is still under dispute^9,10^. In addition, it has been debated that whether autophagy could be induced in cells lacking Bax and Bak^9,10^. If there is autophagy activation in cells lacking Bax and Bak, then what would be the role and function of autophagy? A previous cell line study^9^ has shown that autophagy can be activated in the absence of apoptosis, however, whether autophagy would be activated in the absence of apoptosis and the roles of autophagy in skeletal muscle *in vivo* remains unknown.

To reveal the precise molecular mechanisms by which mechanical loading induces pressure ulcers, we developed a muscle-specific, conditional, double-deficient mouse model (Bax^−/−^Bak^−/−^ mice; Supplementary Figure 1) to examine the role of Bax and Bak in pressure ulcers. Moreover, we conducted an autophagy inhibition study to identify the role of autophagy in mice with different genotypes. Finally, we examined whether mechanical compression could induce autophagy in skeletal muscle deficient for Bax and Bak.

## Results

### Skeletal muscle structural integrity and functions are preserved in Bax^−/−^Bak^−/−^ mice

**Figure 1:**
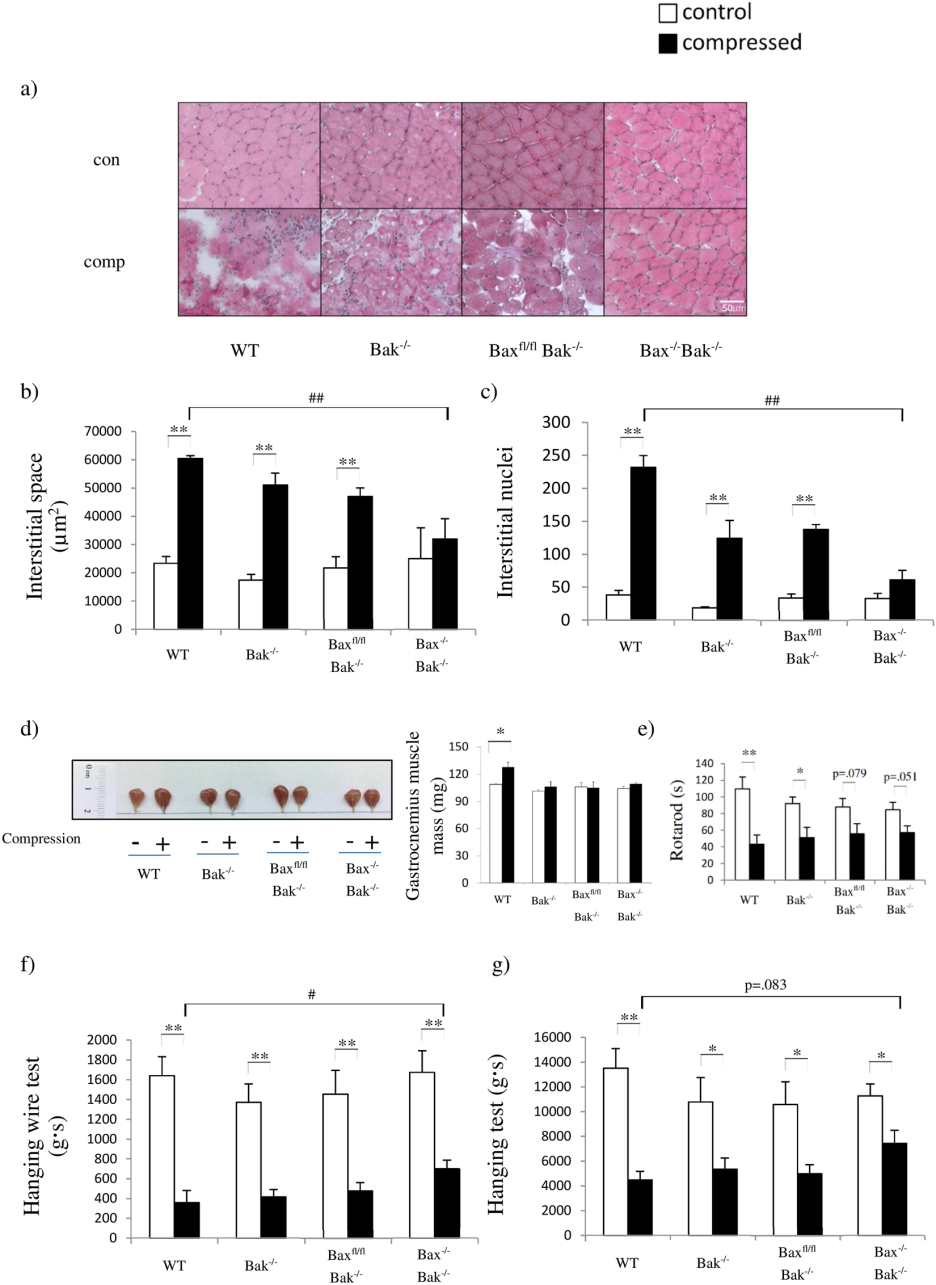
Ablation of Bax and Bak preserves skeletal muscle structural integrity after mechanical compression. (**a**) Representative images of H&E-stained sections from control (con) and compressed (comp) muscle tissues collected from wild-type (WT), Bak^−/−^, Bax^fl/fl^Bak^−/−^, and Bax^−/−^Bak^−/−^ mice. Figures (**b**) and (**c**) show the comparison of animals (n = 3 mice/group; WT, Bak^−/−^, Bax^fl/fl^Bak^−/−^, and Bax^−/−^Bak^−/−^ mice in this order) for (**b**) interstitial space, (**c**) numbers of interstitial nuclei and (**d**) representative images of control (“−” or clear bars) and compressed (“+” or shaded black bars) muscle tissues. (**e**) to (**g**) show the comparison of animals (WT, Bak^−/−^, Bax^fl/fl^Bak^−/−^, and Bax^−/−^Bak^−/−^ mice in this order) for (**e**) rotarod performance (n = 5 mice/group), (**f**) performance in hanging wire test and (**g**) performance in hanging test before (clear bars) and after (shaded bars) compression. All values are expressed as means ± standard error of mean. Pairwise comparison was performed with Student’s t-test, *P < 0.05, **P < 0.01. One-way analysis of variance (ANOVA) and Tukey’s *post hoc* test were used to compare multiple groups, ^##^P < 0.01.

To determine whether muscle structural integrity can be maintained by preventing apoptosis, hematoxylin and eosin (H&E) staining was used to assess skeletal muscle morphology. Our histological analyses revealed that compressed muscles from wild-type (WT), Bak^−/−^, and Bax^fl/fl^Bak^−/−^ mice showed abnormal morphologies (Fig. 1a,b and c). Compressed muscles from Bax^−/−^Bak^−/−^ mice were strikingly resistant to losses in muscle structural integrity in response to prolonged compression (Fig. 1a and d). However, compression only mildly impaired the rotarod performance of Bax^−/−^Bak^−/−^ mice (Fig. 1e). This implies that the maintenance of muscle structural integrity helps animals to partially preserve their physiological performance after compression (Fig. 1f and g). Moreover, the physiological performance in different experiments was deteriorated to different extents owing to the fact that gastrocnemius muscle might be recruited to varied extents in different tests. Of note, we cannot exclude the possibility that the improvement might be partly attributed to reduction in inflammation resulting in alleviation of pain. Although the ablation of Bax and Bak can maintain muscle structural integrity in a short period after mechanical compression, whether this short-term blunting of signaling would negatively affect regeneration or recovery of muscle remains to be investigated.

### Pressure-induced apoptosis and inflammation are mediated by Bax and Bak

**Figure 2:**
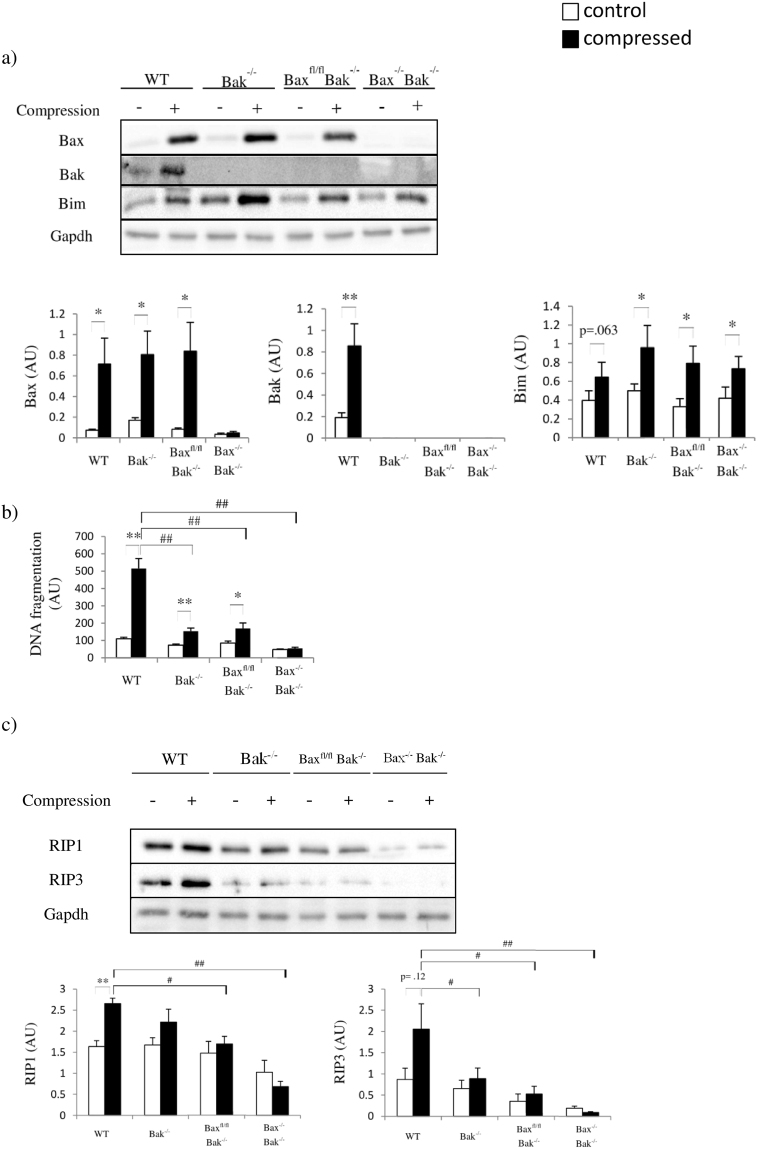
Bax and Bak regulate both apoptotic and necrotic cell death. (**a**) to (**c**) show the comparison of animals (WT, Bak^−/−^, Bax^fl/fl^Bak^−/−^, and Bax^−/−^Bak^−/−^ mice in this order) for (**a**) amounts of Bax, Bak, and Bim proteins by Western blots (n = 6 mice/group), (**b**) DNA fragmentation (n = 6 mice/group) and (**c**) amounts of RIP1 and RIP3 proteins by Western blots in control (“−” or clear bars)and compressed (“+” or shaded black bars) muscle tissues. All values are expressed as means ± standard error of mean. Pairwise comparison was performed with Student’s t-test, *P < 0.05, **P < 0.01. One-way analysis of variance (ANOVA) and Tukey’s post hoc test were used to compare multiple groups, ^#^P < 0.05, ^##^P < 0.01.

**Figure 3:**
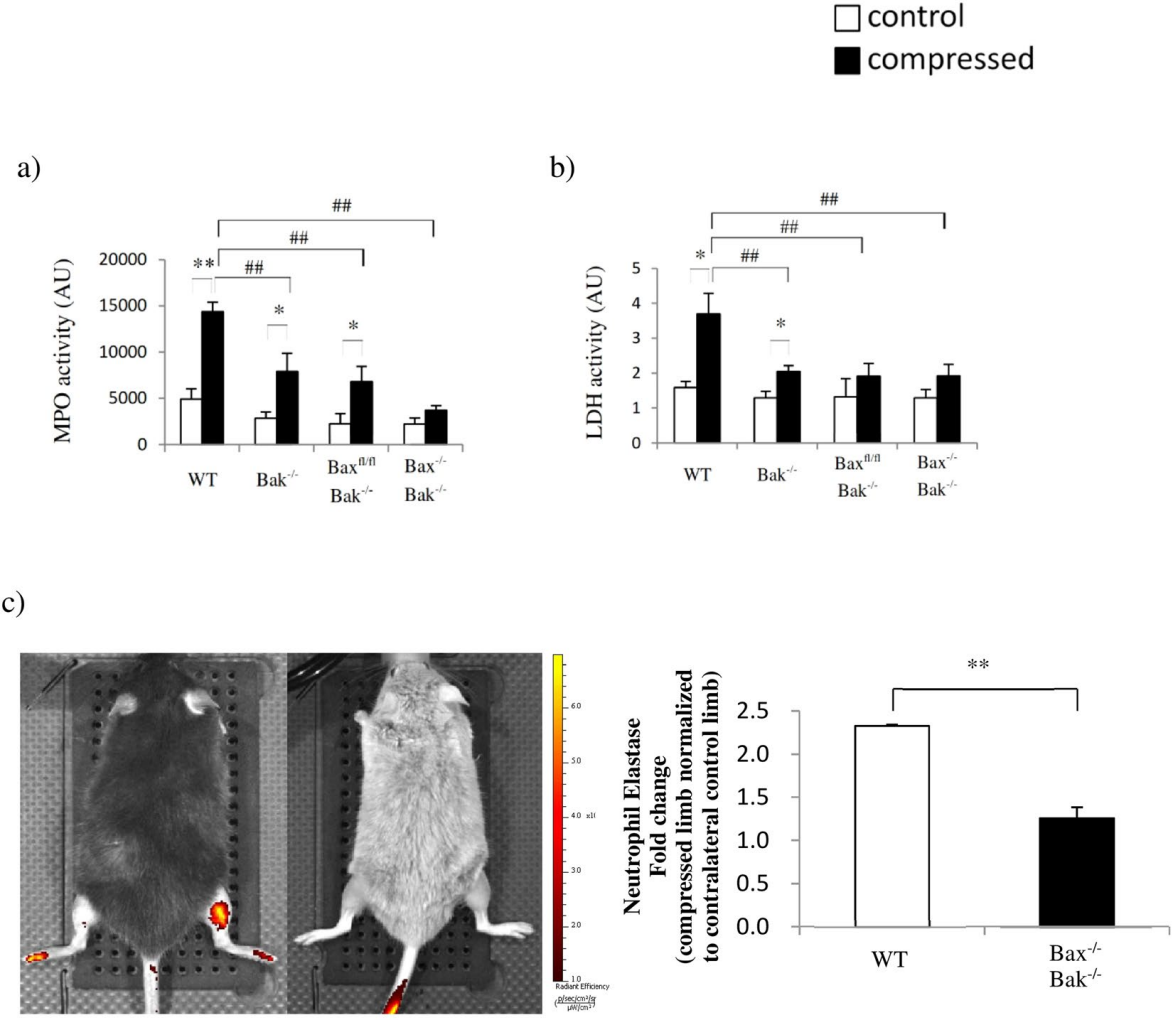
Pressure-induced inflammation in skeletal muscle is mediated by apoptosis. (**a**) to (**b**) show the comparison of animals (WT, Bak^−/−^, Baxfl/flBak^−/−^, and Bax^−/−^Bak^−/−^ mice in this order) for (**a**) myeloperoxidase (MPO) activity (n = 6 mice/group), and (**b**) lactate dehydrogenase (LDH) activity (n = 6 mice/group) in control (“−” or clear bars) and compressed (“+” or shaded black bars) muscle tissues. All values are expressed as means ± standard error of mean. Pairwise comparison was performed with Student’s t-test, *P < 0.05, **P < 0.01. One-wayanalysis of variance (ANOVA) and Tukey’s post hoc test were used to compare multiple groups, ^##^P < 0.01. (**c**) The detection of neutrophil elastase activities after mechanical compression by *in vivo* fluorescence imaging (n = 3 mice/group).

Consistent with our previous findings^4,5^, Bax and Bak were simultaneously upregulated in compressed muscle in WT mice but not in the other mice (Fig. 2a). Next, we performed a cell death detection ELISA to assess the degree of cytoplasmic histone-associated DNA fragmentation. Compression significantly increased DNA fragmentation in the muscles of all of the animals except the Bax^−/−^Bak^−/−^ mice (Fig. 2b). Besides the prevention of muscle apoptosis induction, the Bax and Bak double-deficiency impeded pressure-induced RIP1-RIP3 activation, which is required for necrosis (Fig. 2c)^11^. As RIP1-RIP3 activation was only observed in compressed muscle from the WT, Bak^−/−^, and Bax^fl/fl^Bak^−/−^ mice, we suspected that the apoptotic cells underwent secondary necrosis, which might have induced inflammation and caused tissue swelling in the compressed muscle. Therefore, we examined the enzymatic activities of MPO and LDH in skeletal muscle. MPO and LDH activities increased in the compressed muscle from WT, Bak^−/−^, and Bax^fl/fl^Bak^−/−^ mice, but not from Bax^−/−^ Bak^−/−^ mice, which showed no signs of inflammation (Fig. 3a and b). To confirm whether apoptosis is required for pressure-induced inflammation, *in vivo* imaging was performed in WT and Bax^−/−^Bak^−/−^ mice 2 days after compression (Fig. 3c). We observed that inflammation was not activated in Bax^−/−^Bak^−/−^ mice. In contrast, inflammation was markedly activated in skeletal muscle from WT mice. These results indicate that pressure-induced inflammation in skeletal muscle is significantly modulated by Bax-Bak-mediated apoptosis.

### Mechanical pressure leads to activation of autophagy and disruption of mitochondrial homeostasis

**Figure 4:**
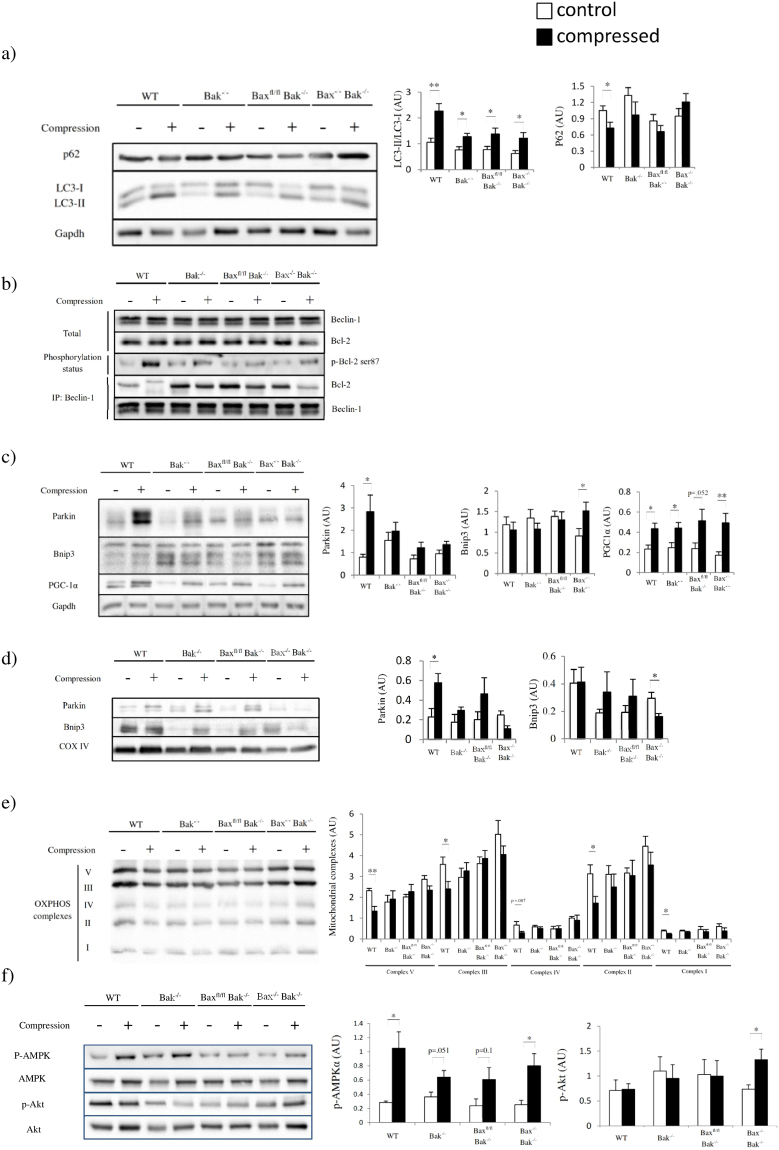
Compression induces degradation of mitochondria by autophagy. (**a**) to (**e**) show the comparison of animals (WT, Bak^−/−^, Bax^fl/fl^Bak^−/−^, and Bax^−/−^Bak^−/−^ mice in this order) by Western blots for (**a**) amounts of p62 and LC3-II/I ratio (n = 6 mice/group), (**b**) Beclin-1, Bcl-2, phosphorylated Bcl-2 at Ser87, immunoprecipitated Bcl-2, and immunoprecipitated Beclin-1 proteins (n = 4 mice/group), (**c**) Parkin, Bnip3 and PGC-1α proteins (n = 6 mice/group), (**d**) mitochondrial Parkin and Bnip3, (**e**) mitochondrial complexes (n = 4 mice/group), including complexes I-V, and (**f**) AMPK and Akt protein phosphorylation status in control (“−” or clear bars) and compressed (“+” or shaded black bars) muscle tissues. Pairwise comparison was performed with Student’s t-test, *P < 0.05, **P < 0.01.

We next studied autophagy in compressed muscle. The LC3-II to LC3-I ratio and the LC3-II and total LC3 abundance increased after compression in most of the animals (Fig. 4a and Supplementary Figure 2a and b). However, the abundance of p62 decreased only in the compressed muscle from WT mice (Fig. 4a). Therefore, we examined the interaction between Bcl-2 and Beclin-1 because their dissociation is essential for the activation of autophagy in response to cellular stress. Our immunoprecipitation results indicated that compression led to Bcl-2 and Beclin-1 dissociation although their abundances remained unchanged (Fig. 4b and Supplementary Figure 2c and d). Detachment was induced by Bcl-2 phosphorylation (Fig. 4b and Supplementary Figure 2e). We suspected that autophagy activation targeted mitochondria for degradation as ischemia-reperfusion injury has been shown to cause mitophagy in different tissues^12^. Our immunoblot results showed that the mitophagy protein Parkin, both cytoplasmic fraction and mitochondrial fraction, was upregulated after compression (Fig. 4c and d) in WT mice. Notably, Parkin was not activated in Bax^−/−^Bak^−/−^ muscle, suggesting that the mitochondrial membrane potential in Bax^−/−^Bak^−/−^ muscle might not be low enough to recruit Parkin and initiate mitophagy recycling of deteriorated mitochondria^13^. In contrast, Bnip3 was upregulated in the cytoplasmic fraction and, surprisingly, downregulated in the mitochondrial fraction. As Bnip3 did not insert in the mitochondrial membrane, it should not induce mitophagy in Bax^−/−^Bak^−/−^ mice. As compression altered the expression of mitochondrial proteins in WT and Bax^−/−^Bak^−/−^ mice, we examined the protein abundance of PGC-1α and the status of mitochondrial OXPHOS (complexes I–V) to determine the state of mitochondrial homeostasis in compressed muscle (Fig. 4c,d and e). Our results revealed that compression induced both mitochondrial biogenesis and mitophagy in compressed muscle; however, the abundance of the mitochondrial complexes decreased in compressed muscle from WT mice only. These results suggest that autophagy was over-activated and that disequilibrium between mitophagy and mitochondrial biogenesis might exacerbate muscle cell death in WT mice. However, whether mitophagy and mitochondrial biogenesis are linked processes or independent events remains to be answered. Autophagy seems to be carefully regulated in mice with different genotypes by upstream signaling pathways (Fig. 4e). These results prompted us to define the nature of autophagy in compressed muscle.

### Inhibition of autophagy prevents pressure-induced degradation of mitochondria

**Figure 5:**
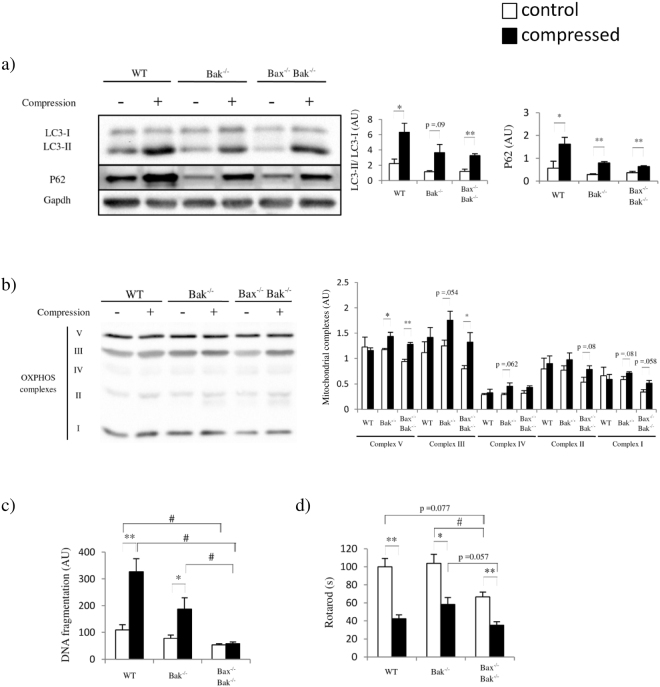
Inhibition of autophagy preserves mitochondrial contents and ameliorates cell death in compressed skeletal muscle. (**a**) to (**d**) show the comparison of animals (n = 4 mice/group; WT, Bak^−/−^, and Bax^−/−^Bak^−/−^ mice in this order) injected with the autophagy inhibitor colchicine for (**a**) amounts of p62 and L3C-I/II proteins by Western blots, and (**b**) mitochondrial complexes, including complexes I-V, by Western blots, (**c**) DNA fragmentation, and (**d**) rotarod performance in control (“−” or clear bars) and compressed (“+” or shaded black bars) muscle tissues. All values are expressed as means ± standard error of mean. Pairwise comparison was performed with Student’s t-test, *P < 0.05, **P < 0.01. One-way analysis of variance (ANOVA) and Tukey’s post hoc test were used to compare multiple groups, ^#^P < 0.05.

We conducted an autophagy inhibition study in our animals using colchicine to define the (pro-survival or pro-death) role of autophagy in PI. The colchicine was used because it is a well-established inhibitor of microtubule-mediated transport of autophagosome to lysosome in animal studies^14–16^. Although colchicine has been shown to be anti-inflammatory in human^17,18^ and in animals^19,20^, the dose we adopted is much lower than the dose demonstrated to be anti-inflammatory in other animal studies. Colchicine inhibited autophagy as evidenced by the accumulation of LC3-II and p62 (Fig. 5a and Supplementary Figure 3)^15,21^. Inhibition of autophagy increased the mitochondrial contents in Bak^−/−^ and Bax^−/−^Bak^−/−^ mice, but not in WT mice (Fig. 5b). To further determine whether inhibition of autophagy would ameliorate cell death in compressed muscle, we performed a cell death ELISA for DNA fragmentation analysis. Our results showed that compression increased DNA fragmentation by about 300% in WT mice with inhibited autophagy (Fig. 5c). Of note, compression increased DNA fragmentation by about 500% in WT mice without inhibition of autophagy (Fig. 2b). These results reflect that autophagy could contribute to cell death in compressed muscle from WT mice. In Bax^−/−^Bak^−/−^ mice, inhibition of autophagy did not alter DNA fragmentation; however, it led to both increase in mitochondrial contents and further decrease in rotarod performance (Fig. 5d) when compared to WT (Fig. 1e). It has been demonstrated that inefficient removal of malfunctioning mitochondria negatively affects the physical performance of animals^22,23^. Therefore, we suspect that the inhibited autophagy leading to inefficient removal of mitochondria might further deteriorate rotarod performance in Bax^−/−^Bak^−/−^ mice.

## Discussion

In the present study, we showed that ablation of Bax and Bak protected skeletal muscle against PI. Compression consistently deteriorated muscle histology and impaired the overall motor coordination in animals regardless of their genotypes, which is consistent with observations from human studies^24^. Interestingly, muscle histology and part of the physiological performance of skeletal muscle were maintained in Bax^−/−^Bak^−/−^ mice after compression. Moreover, the muscle apoptotic cell death programme was not executed in Bax^−/−^Bak^−/−^ mice after compression. This study is the first to demonstrate that skeletal muscle cells that were deficient of Bax and Bak were strikingly resistant to mechanical compression injury. Cells that are deficient of both Bax and Bak develop resistance to apoptosis has been demonstrated in different tissues such as blood and neural tissue^25,26^. Deletion of Bax and Bak in neural tissue represents a possible therapeutic strategy for amyotrophic lateral sclerosis^25^. Here, ablation of Bax and Bak in skeletal muscle was shown to prevent the PI-induced apoptosis. In addition, inflammation and necrosis were not activated in Bax^−/−^Bak^−/−^ mice after compression, implying that the compression-induced necrosis and, hence, inflammation were mediated by apoptosis. The apoptotic cells in WT mice might not be efficiently removed by phagocytes, and therefore, the dying cells progressed to secondary necrosis^27^, however, this speculation needs to be further investigated. It would be interesting to examine if the leaked contents from necrotic cells would provoke inflammation by upregulating MPO and LDH activities in the compressed muscle. Besides inflammation in compressed muscle, a recent study has shown that mechanical pressure leads to morphological changes and inflammation in human ischemic skin^28^. However, apoptosis was not found to be activated in the compressed skin while another study has suggested that sustained tissue deformations elevate intracellular calcium ions which lead to cell death^29^. Although compressed muscle and skin share similar pathological changes (e.g., activation of inflammation and altered morphologies), whether these changes are mediated by the same molecular pathways (e.g., apoptosis and autophagy) remains to be answered.

The activation of autophagy was observed in mice with different genotypes, however, the degree of activation of autophagy varied. Although the LC3-II to LC3-I ratio increased across different groups, p62 was only decreased in WT mice. We speculated that compression induced autophagy to the greatest extent in WT mice. In line with this observation, mitochondrial content was only decreased in compressed muscle from WT mice, suggesting that autophagy targeted and recycled mitochondria. The mitophagy protein Parkin, which was only upregulated in compressed muscle from WT mice, should play a major role in promoting both autophagy and apoptosis in WT mice^30^. Mitochondrial depolarization promotes Parkin-mediated mitophagy^31^. Parkin was upregulated in WT mice only, suggesting that the ablation of bak and ablation of bax and bak might protect skeletal muscle against mitochondrial depolarization. At the same time, it has been observed that cytoplasmic and mitochondrial Bnip3 were upregulated and downregulated in Bax^−/−^Bak^−/−^ mice respectively. The upregulation of cytoplasmic Bnip3 might serve as an alternative mechanism to activate excessive autophagy leading to cell death in muscle without apoptosis^32^. Moreover, Bnip3 has been regarded as an autophagy receptor for targeted removal of mitochondria. Therefore, the decreased mitochondrial Bnip3 might imply that the activated autophagy was not targeting mitochondria, instead, it occurred in a more generalized fashion in removing damaged organelles and other cellular components. Alternatively, the decreased mitochondrial Bnip3 might imply the damaged mitochondria had been targeted for autophagy and had already been removed before the selected time point (i.e., 48 hours). Therefore, the net remaining mitochondria were healthy and no longer targeted by Bnip3 for autophagy. Hence, mitochondrial Bnip3 was decreased in Bax^−/−^Bak^−/−^ mice. Intriguingly, we detected a significant increase in Akt phosphorylation and unaltered mitochondrial content in Bax^−/−^Bak^−/−^ mice, suggesting that Akt might serve as an autophagy inhibitor counteracting the possible detrimental effects of Bnip3^33,34^.

There has been a debate over the role of autophagy in different diseases^35–37^. In the current study, we have demonstrated that autophagy performs dual roles depending on the genotypes. Our results suggest that autophagy plays a pro-death role in WT mice and a pro-survival role in Bax^−/−^Bak^−/−^ mice. Excessive autophagy/mitophagy contributes to cell death in WT mice^38,39^. The inhibition of autophagy could partially alleviate cell death in WT mice, however, it did not improve the physiological performance of WT mice. In fact, the inhibition even deteriorated the physiological performance of Bax^−/−^Bak^−/−^ mice. These results give insights to the role of autophagy. It has been previously reported that blocking apoptosis induces autophagy which is important in recycling intracellular impaired organelles and protein aggregates^25^ and maintaining physiological performance in skeletal muscle^40^. In line with these previous findings, we demonstrate that autophagy is important in maintaining muscle performance and, furthermore, in regulating the mitochondrial homeostasis. We show that the over-activation of autophagy results in exacerbated cell death and inhibition of autophagy can alleviate cell death. These findings are particularly important for understanding diseases related to uncontrolled activation of autophagy. A limitation of the current study is that we do not know whether autophagy occurs prior to apoptosis, therefore, further investigation is needed to determine the sequence of autophagy and apoptosis.

Taken together, our results indicate that PI pathogenesis is mediated by Bax-Bak-dependent apoptosis. Ablation of Bax and Bak prevents RIP1-RIP3 activation and subsequent inflammation in compressed muscle. These findings are the first to uncover the detrimental role of excessive autophagy in PI. Inhibiting Bax and Bak and manipulating autophagy could be useful strategies for preventing/treating apoptosis- and inflammation-related muscle diseases such as pressure ulcers. Further investigation is needed to examine whether manipulating apoptosis and autophagy could alleviate other muscle injuries such as blunt trauma, cardiotoxin-induced injury and other types of reperfusion injury.

## Materials and Methods

### Animal care

The following mouse lines were used in our experiments: (1) C57Bl/6 (wild-type, WT), (2) Bax^tm2Sjk^Bak1^tm1Thsn^ (Bax^fl/fl^Bak^−/−^), and (3) HSA-MCM (skeletal muscle-specific inducible Cre transgenic mouse strain). Bax^fl/fl^Bak^−/−^ mice were purchased from Jackson Laboratories (6329, Hancock, ME, USA). HSA-MCM mice were kind gifts donated by Dr John McCarthy and Dr Karyn Esser (University of Kentucky). The WT, Bak^−/−^, and Cre^+/−^ Bax^fl/fl^Bak^−/−^ mice were bred and housed in the same humidity- and temperature-controlled environment and exposed to a 12-hour light/12-hour dark cycle in the Centralized Animal Facilities at The Hong Kong Polytechnic University. To generate Cre^+/−^ Bax^fl/fl^Bak^−/−^ mice, HSA-MCM mice and Bax^fl/fl^Bak^−/−^ were allowed to mate to give Cre^+^ Bax^+/fl^Bak^+/−^ mice. Cre^+^ Bax^+/fl^Bak^+/−^ mice and Bax^fl/fl^Bak^−/−^ were allowed to mate to generate Cre^+^ Bax^fl/fl^Bak^−/−^ mice. To induce double-deficiency (Bax^−/−^Bak^−/−^ mice), Cre^+^ Bax^fl/fl^Bak^−/−^ mice were intraperitoneally injected with tamoxifen dissolved in corn oil (Sigma, St. Louis, MO, USA) for 5 consecutive days. Mice were allowed access to standard animal diets and water ad libitum. Male 20–24-week-old mice were used in this study.

### Study approval

Experiments were approved by the Animal Subjects Ethics Sub-committee (ASESC), The Hong Kong Polytechnic University. All methods were confirmed to be performed in accordance with the relevant guidelines and regulations.

### Experimental design

Prior to muscle compression, mice were anesthetized with ketamine and xylazine via intraperitoneal injection. The optimized compression model was applied to WT, Bak^−/−^, Bax^fl/fl^Bak^−/−^, and Bax^−/−^Bak^−/−^ mice. A static pressure of 100 mm Hg of compression load was applied over the right gastrocnemius muscles of the animals. Compression was applied using a motorized, computer-controlled, mechanical indenter, and the magnitude was monitored continually by a three axial transducer. The compression duration was 4 hours. Contralateral limbs served as controls for the compression experiment. Physiological measurements were conducted 46 hours after compression and animals were sacrificed 2 hours after physiological measurements via CO_2_ ventilated euthanasia. Gastrocnemius muscle tissues from the hind limb were weighed and snap frozen with isopentane in liquefied nitrogen and stored at −80 °C. The frozen tissues, which were embedded in optimal cutting temperature compound (Sakura, CA, USA), were sectioned and used for histological analysis and apoptotic assessment^4,5^. *In vivo* imaging was used to examine the activity of neutrophil elastase in the second batch of animals. The *in vivo* imaging was conducted 42 hours after compression. The autophagy inhibition study was conducted in the third batch of animals.

### Muscle morphology

H&E staining was used to demonstrate the histology of the skeletal muscle in comparative experimental models. Ten-micrometer-thick frozen muscle cross-sections were cut perpendicular to the skin in a freezing cryostat at −20 °C, fixed with 10% formalin, and then air-dried for 10 minutes at room temperature. Routine histological examinations were performed on the cross sections using H&E staining (Sigma, St. Louis, MO, USA). Nuclei in the interstitial space and the area of the interstitial space were assessed. Nuclei were counted in three random fields, and the average numbers of nuclei were reported.

### Rotarod running

The rotarod test was used to examine the muscle condition, coordination, and balancing ability of the mice^41^. Mice were placed on a rotating tube. The tube initially rotated at a speed of 5 rpm. When the mice started to run, the tube accelerated from 5 to 20 rpm gradually within the first minute, after which it maintained that speed. The average running time was used as an outcome measure.

### Hanging wire test

The hanging wire test was used to monitor muscle function over time^41^. Briefly, a 55-cm-wide, 2-mm-thick metal wire was fixed at two vertical stands. Mice were allowed to grasp the middle of the wire with their fore limbs and turn upside-down along the wire axis. A timer was started when the mouse was grasping the wire with its four paws. Each holding time was recorded. The average holding time was obtained from three trials. The holding impulse [mass (gram) × hang time (sec)] was used as an outcome measure.

### Four limb hanging tests

The four limb hanging test was used to monitor muscle strength and condition over time^41^. Briefly, a 1 cm × 1 cm square grid was placed 25 cm above soft bedding. The mouse was placed on the grid so that it grasped the grid with its four paws. A timer was started when the grid was inverted. Each holding time was recorded. The average holding time was obtained from three trials. The holding impulse was used as an outcome measure.

### Western blot analyses

Protein abundances of the apoptotic factors were measured. Protein extracts were boiled at 95 °C for 5 minutes in Laemmli buffer with 5% β-mercaptoethanol (Bio-Rad, Hercules, CA, USA). Fifty-microgram protein samples were loaded onto 10–15% polyacrylamide gels. After electrophoretic separation by sodium dodecyl sulphate-polyacrylamide gel electrophoresis (SDS-PAGE) the proteins were transferred to PVDF membranes (Millipore, Darmstadt, Germany). Equal loading and transfer efficiency was verified by staining gels with Coomassie blue and staining membranes with Ponceau S red. After transferring, the membranes were blocked in 5% non-fat milk or 5% BSA in Tris-buffered saline with 0.1% Tween-20 (TBST) for 1 hour at room temperature. Then, the membranes were incubated overnight at 4 °C with the corresponding primary antibody in TBST with 2% BSA. Total OXPHOS Western blot antibody cocktail (ab110413) was purchased from Abcam (Cambridge, MA, USA). LC3B (#2275), p62 (#5114), Bnip3 (#3769), Akt (#4691), p-Akt (#4056), AMPK (#2532), and p-AMPK (#2535) antibodies were purchased from Cell Signaling (Danvers, MA, USA). Bax (sc-493), Bak (sc-832), Beclin-1 (sc-11427), Bcl-2 (sc-7382), p-Bcl-2 (sc-16323), Parkin (sc-32282), PGC-1α (sc-13067), RIP1 (sc-7881), RIP3 (sc-135170), JNK (sc-571), and p-JNK (sc-6254) antibodies were purchased from Santa Cruz (Santa Cruz, CA, USA). After incubation, membranes were washed three times with TBST for 10 minutes each. After washing, the membranes were incubated with horseradish peroxidase (HRP)-conjugated secondary antibodies at room temperature for 1 hour (1:4000 dilution; 7076 for anti-mouse IgG antibody and 7074 for anti-rabbit IgG antibody, Cell Signaling Technology, Danvers, MA, USA). The Luminata Forte Western HRP substrate (Merck Millipore, Darmstadt, Germany) for HRP chemiluminescence detection was added to the membranes before exposure under a ChemiDoc (Bio-Rad). The resulting bands were quantified by the optical density (OD) × band area and expressed as arbitrary units. Glyceraldehyde 3-phosphate dehydrogenase (GAPDH) was used as an internal control protein. The data were normalized to the GAPDH level unless otherwise specified.

### Co-immunoprecipitation from muscle tissue

Lysate extracts from compressed muscle (gastrocnemius muscles) were subject to immunoprecipitation with an anti-Beclin-1 antibody. The eluates were separated by SDS-PAGE and detected with anti-Bcl-2 and anti-Beclin-1 antibodies^42^.

### Autophagy inhibition study

Animals were treated with colchicine (0.4 mg/kg/day, Sigma, St. Louis, MO, USA) via intraperitoneal injection for two days^21^.

### ***In vivo*** imaging

Images were obtained according to the standard operating procedures provided by the manufacturer. Forty-two hours after compression, mice were injected intravenously with Neutrophil Elastase 680 FAST Fluorescent Imaging Agent (NEV11169, PerkinElmer, Waltham, MA, USA), which is selectively activated by elastase produced by neutrophil cells during inflammation. The hind limbs were imaged using the IVIS® SpectrumCT preclinical *in vivo* imaging system (PerkinElmer, Waltham, MA, USA) 4 hours after agent injection.

### Cell death ELISA

A cell death detection ELISA (11544675001, Roche, Basel, Switzerland) was used to detect mono- and oligonucleosomes in the cytoplasmic fraction of cell lysates. The assay was performed according to the manufacturer’s instructions. A wavelength of 490 nm was used as the reference wavelength.

### Enzymatic activity assay

A myeloperoxidase (MPO) mouse ELISA kit (ab155458, Abcam, Cambridge, MA, USA) and a lactate dehydrogenase (LDH) assay kit (ab102526, Abcam, Cambridge, MA, USA) were used to quantify the enzymatic activities of MPO and LDH, respectively, in muscle lysate. The assays were performed according to the manufacturer’s instructions.

### Statistical analyses

All of the values are presented as means ± SEM. Two-way ANOVA was used to identify the effect of compression. The effect was analyzed using Student’s t test for the control limb and compressed limb analyses for each genotype. One-way ANOVA followed by Tukey’s post hoc test were used to compare variables collected from more than two groups. A value of P < 0.05 was considered statistically significant.

## Electronic supplementary material

Supplementary Figures
