# Supplementary material for: Ablation of Bax and Bak protects skeletal muscle against pressure-induced injury
Source: Sci Rep. 2018 Feb 27;8:3689. doi: 10.1038/s41598-018-21853-5 (PMC5829134; doi:10.1038/s41598-018-21853-5)

Ablation of Bax and Bak protects skeletal muscle against pressure-induced injury

Bjorn T. Tam[1#], Angus P. Yu[2#], Eric W. Tam[3], Douglas A. Monks[4], Xu P. Wang[1,5], Xiao M. Pei[1], Su P. Koh[1], Thomas K. Sin[1,6], Helen K.W. Law[1], Felix N. Ugwu[1], Rashmi Supriya[1], Benjamin Y. Yung[1], Shea P. Yip[1], S. C. Wong[1], Lawrence W. Chan[1], Christopher W. Lai[1], Pin Ouyang[7], and Parco M. Siu[2]*

(# these authors contributed equally)

[1]Department of Health Technology and Informatics, Faculty of Health and Social Sciences, The Hong Kong Polytechnic University, Hong Kong, China

[2]School of Public Health, Li Ka Shing Faculty of Medicine, The University of Hong Kong, Hong Kong, China

[3]Interdisciplinary Division of Biomedical Engineering, Faculty of Engineering, The Hong Kong Polytechnic University, Hong Kong, China

[4]Department of Cell and Systems Biology & Department of Psychology, University of Toronto, Canada

[5]The Key Laboratory of Cardiovascular Remodelling and Function Research, Chinese Ministry of Education and Chinese Ministry of Health, Qilu Hospital, Shandong University, Jinan, Shandong, China

[6]Department of Integrative Biology and Pharmacology, The University of Texas Health Science Center at Houston, Houston, Texas, USA

[7]Department of Anatomy & Transgenic Mouse Core Laboratory, Chang Gung University, Taiwan

Supplementary Figure 1 – 3

Supplementary Figure 1

a)

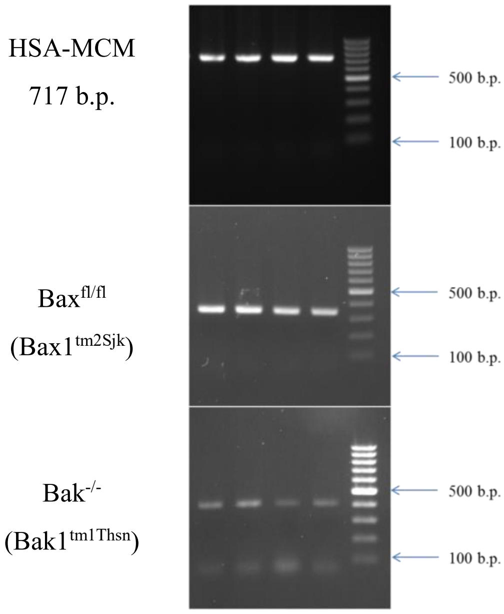

b)

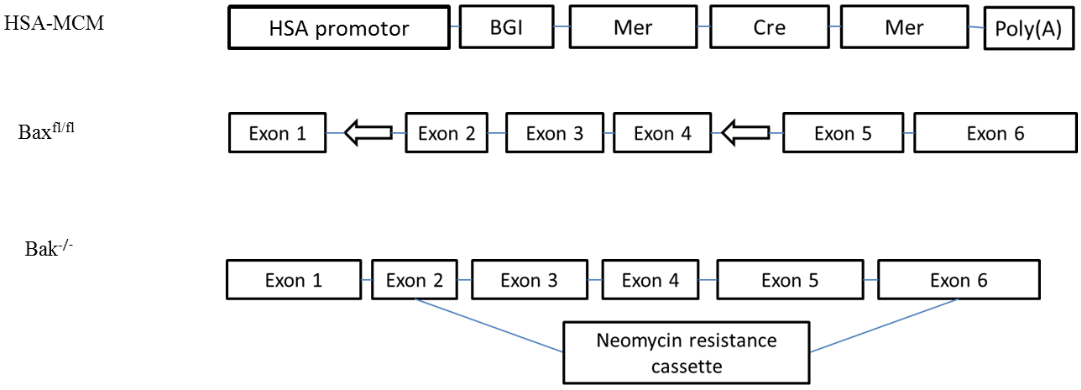

Supplementary Figure 1

(a) Genotyping results – the PCR products observed were as follows: 717 b.p. for Cre^{+}, 340 b.p. for $Bax^{fl/fl}$ and 400 b.p. for $Bak^{-/-}$. (b) Schematic depiction of three transgenic cassettes. HSA-MerCreMer (HSA-MCM) mice express MerCreMer double fusion protein under the regulation of the promotor - human *ACTA1*. When mice possessing loxP-flanked sequences are bred with mice containing HSA-MCM, tamoxifen can be used to induce skeletal muscle-specific deletion of the floxed sequences in their offspring. Two loxP sites were used to target the deletion of exons 2-4 of Bax gene. A neomycin resistance cassette was used to replace exon 2-6 of Bak gene.

Supplementary Figure 2

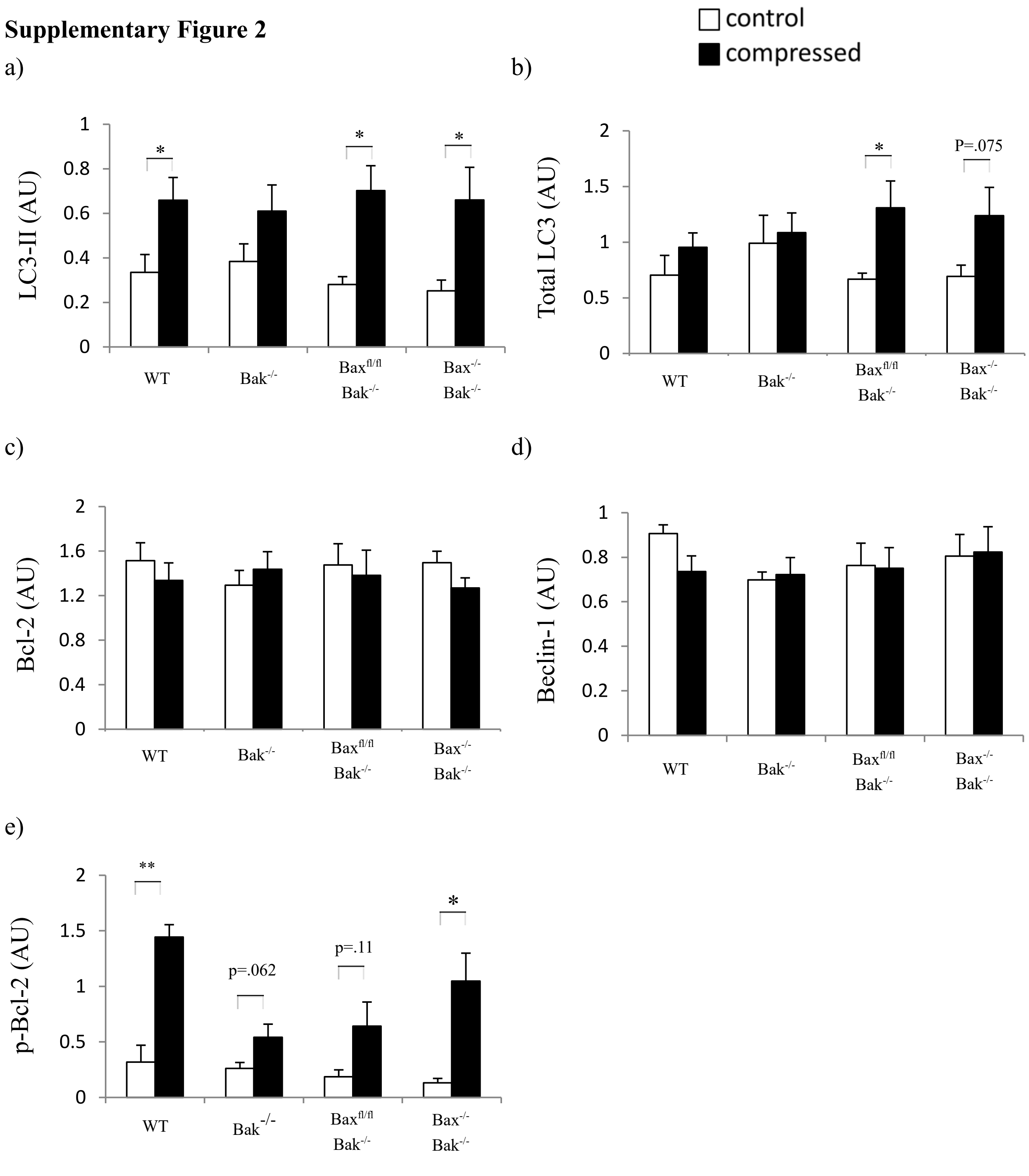

Supplementary Figure 2

Quantification of (a) LC3-II, (b) total LC3, (c) Bcl-2, (d) Beclin-1 and (e) phosphorylated Bcl-2 proteins in control (clear bars) and compressed (shaded bars) muscle tissues collected from WT, $Bak^{-/-}$, $Bax^{fl/fl}Bak^{-/-}$, and $Bax^{-/-}Bak^{-/-}$ mice (n = 4 mice/group). All values are expressed as means ± standard error of mean. Pairwise comparison was performed with Student's t-test, *P<0.05, **P<0.01.

Supplementary Figure 3

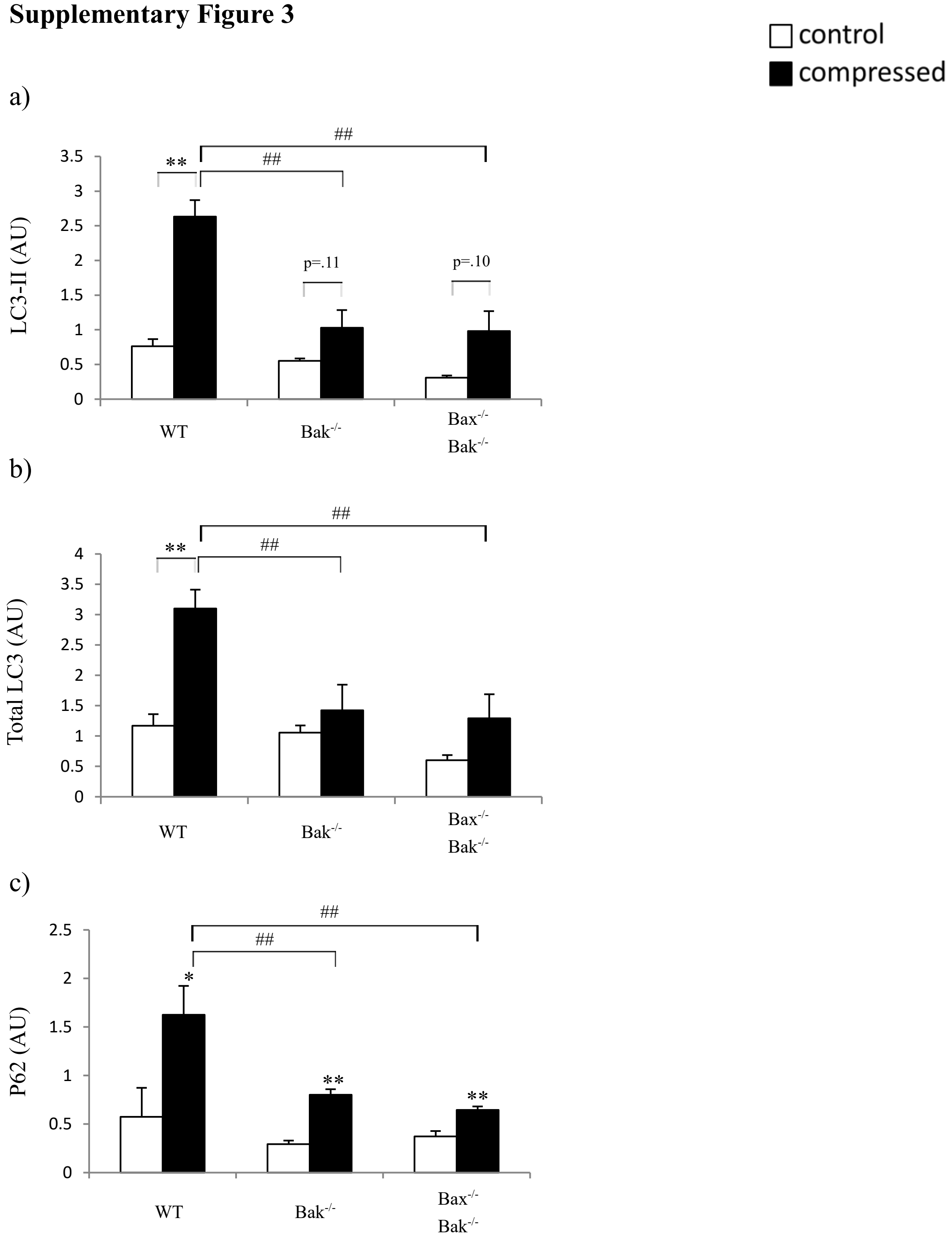

Supplementary Figure 3

Quantification of (a) LC3-II, (b) total LC3, and (c) p62 proteins in control (clear bars) and compressed (shaded black bars) muscle tissues collected from WT, $Bak^{-/-}$, and $Bax^{-/-}Bak^{-/-}$ mice (n = 4 mice/group). All values are expressed as means ± standard error of mean. Pairwise comparison was performed with Student's t-test, *P<0.05, **P<0.01. One-way analysis of variance (ANOVA) and Tukey's *post hoc* test were used to compare multiple groups, $^{\#\#}P<0.01$.

Figure 2a (Raw immunoblots shown in Figure 2a. Samples were run on separate gels and blotted on two membranes for antibody probing. A black solid line is presented to separate two membranes)

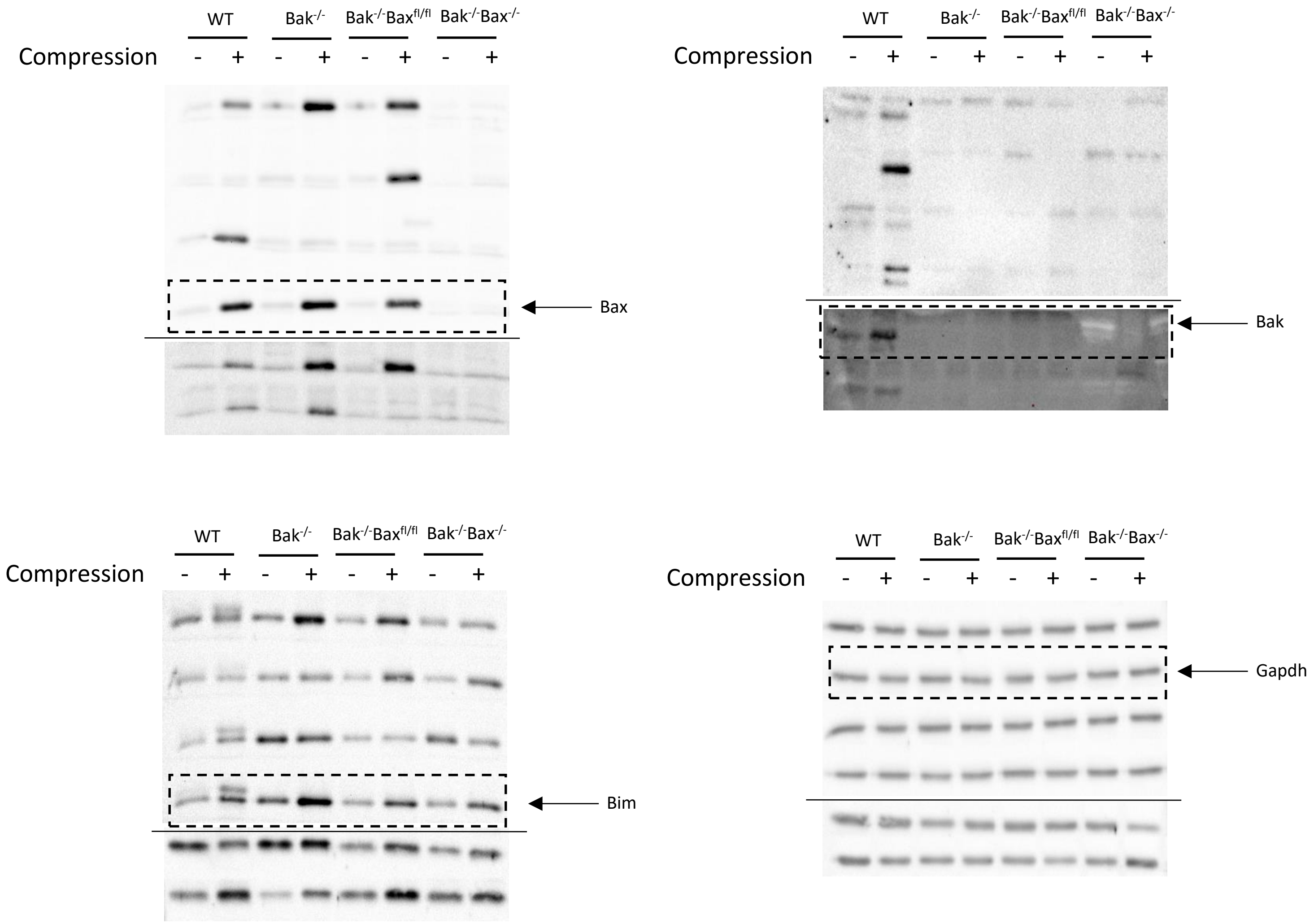

Figure 2c (Raw immunoblots shown in Figure 2c. Samples were run on separate gels and blotted on the same membrane for antibody probing)

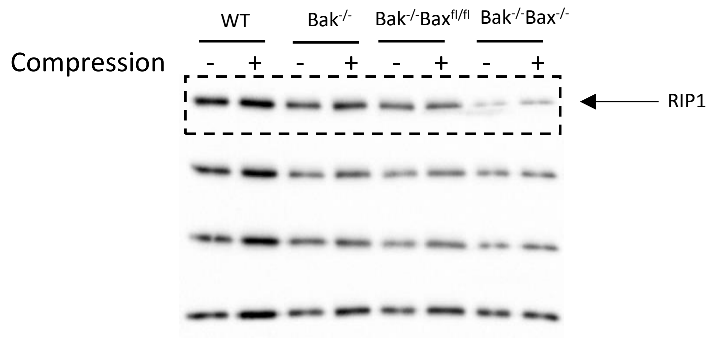

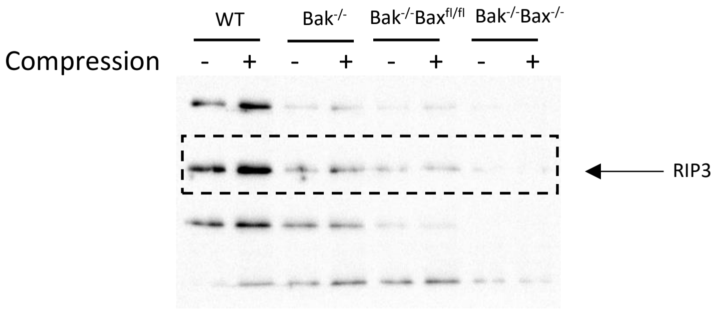

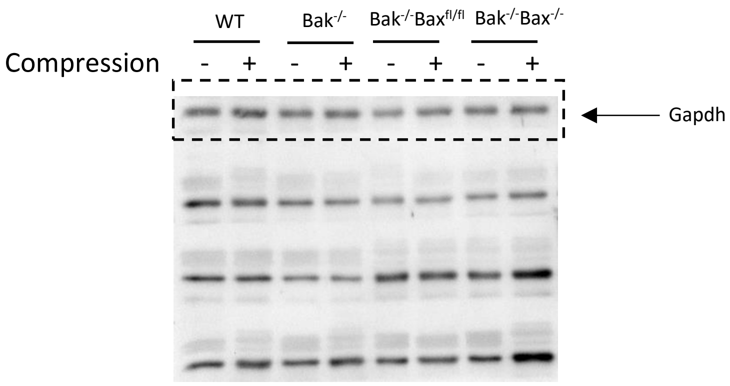

Figure 4a (Raw immunoblots shown in Figure 4a. Samples were run on separate gels and blotted on two membranes for antibody probing. A black solid line is presented to separate two membranes)

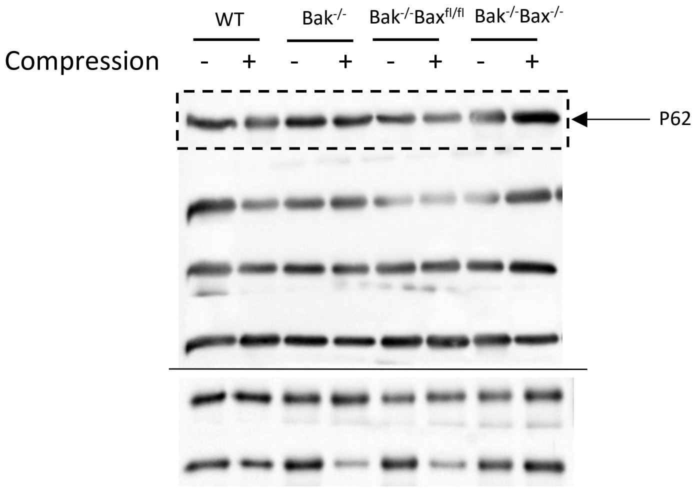

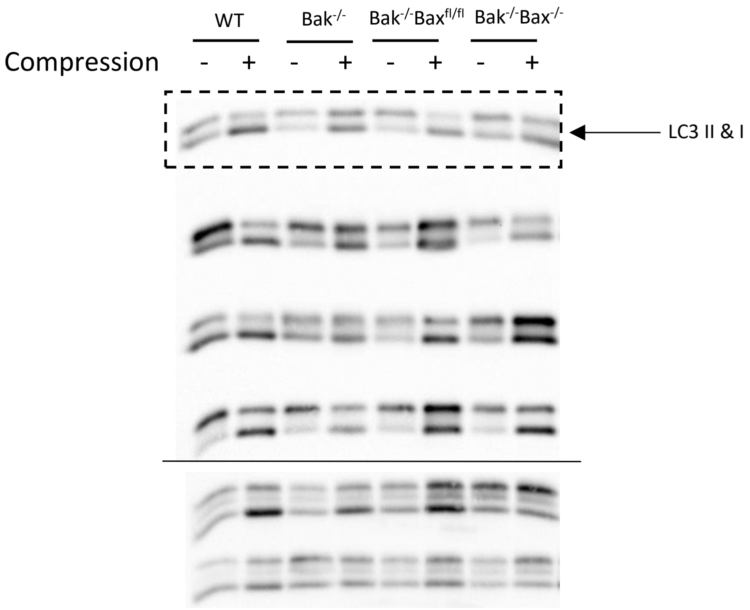

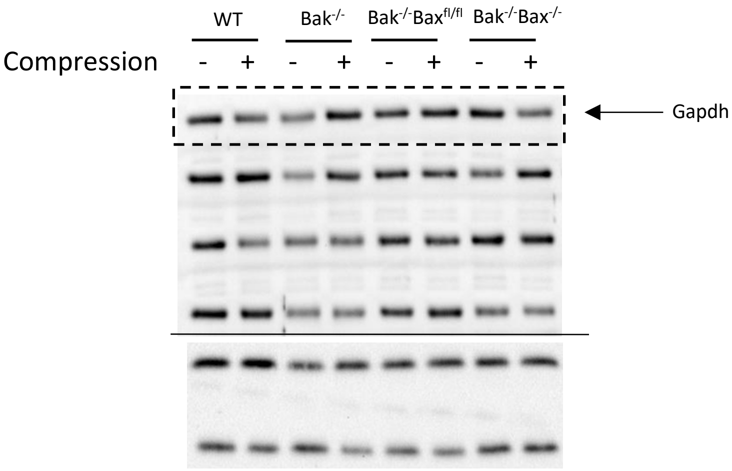

Figure 4b (Raw immunoblots shown in Figure 4b. Samples were run on separate gels and blotted on the same membrane for antibody probing)

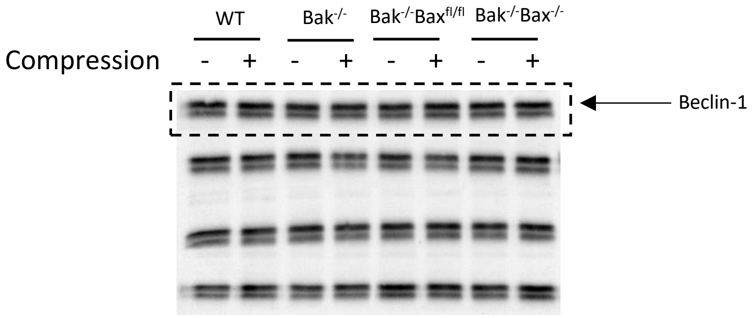

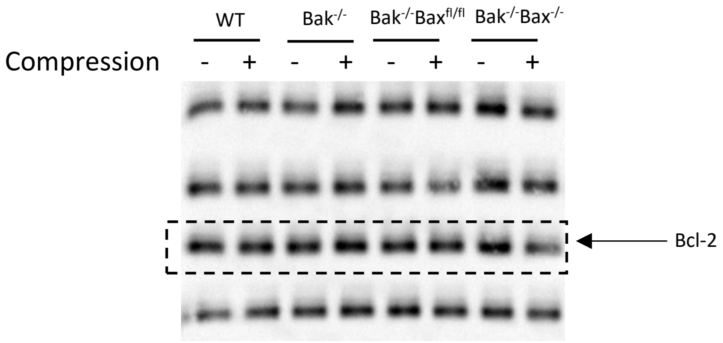

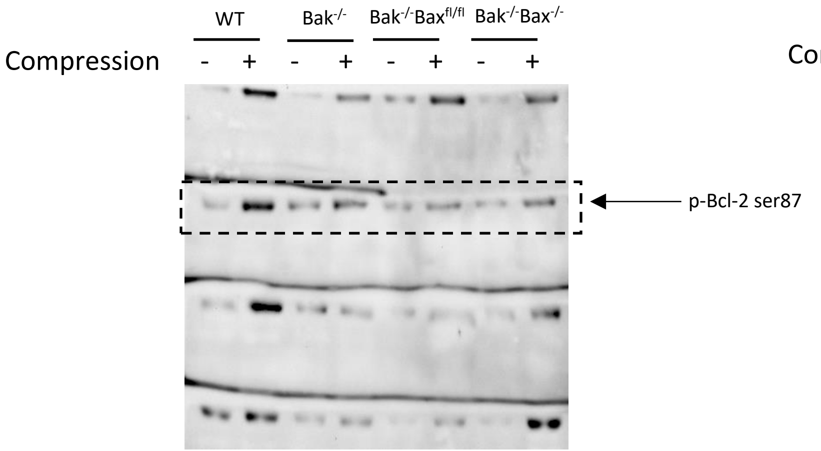

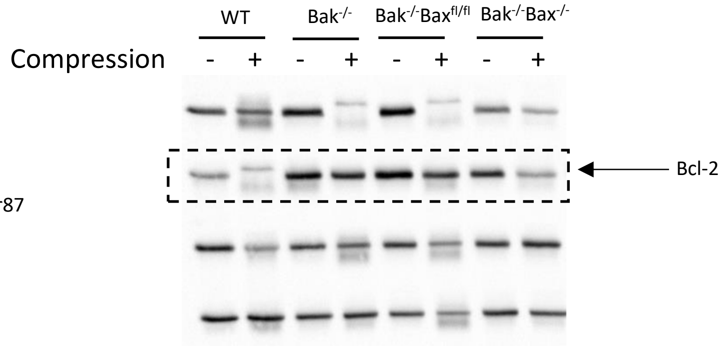

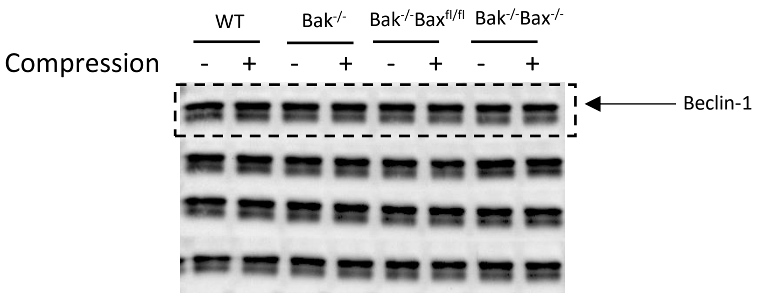

Figure 4c (Raw immunoblots shown in Figure 4c. Samples were run on separate gels and blotted on two membranes for antibody probing. A black solid line is presented to separate two membranes)

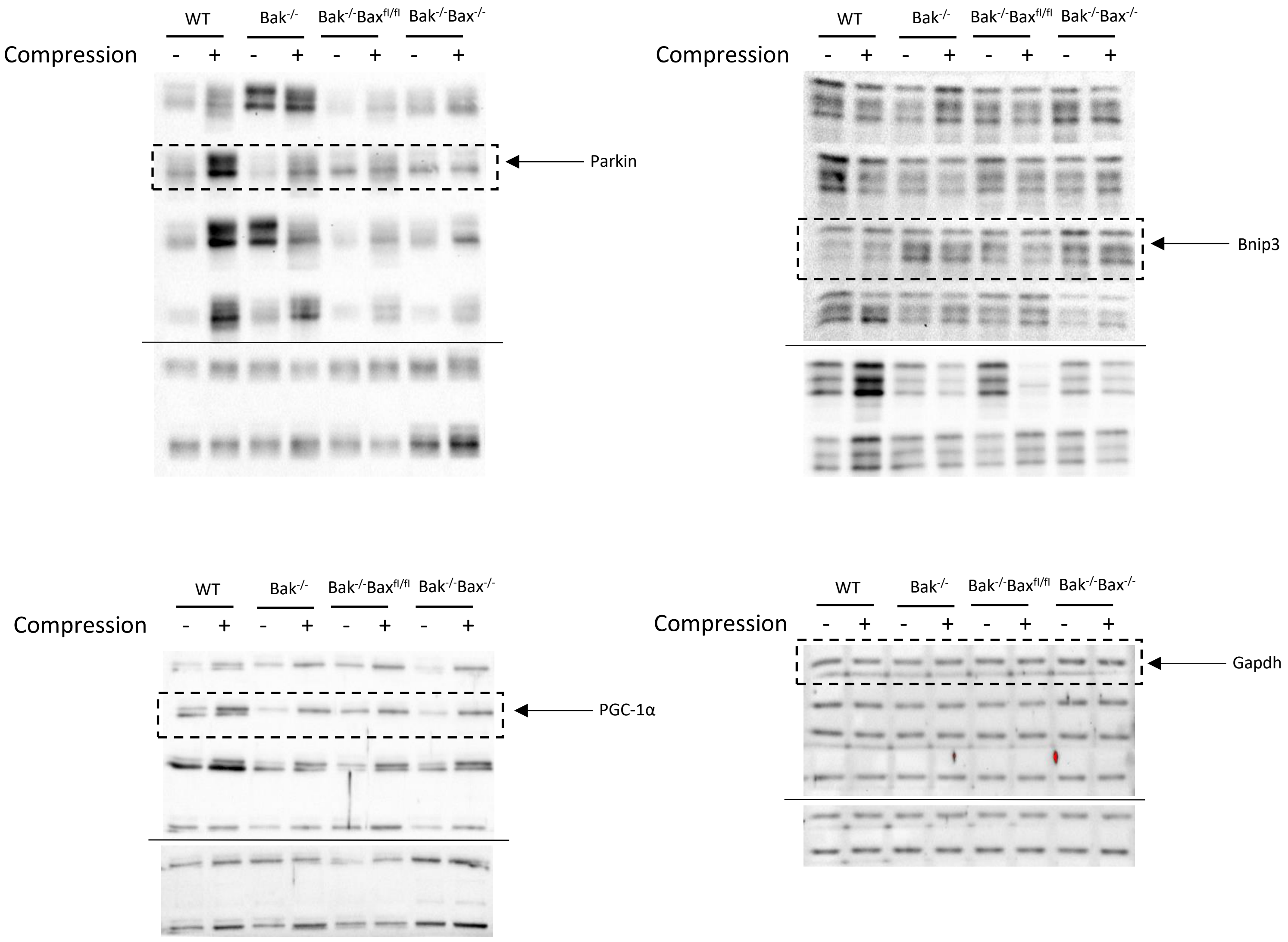

Figure 4d (Raw immunoblots shown in Figure 4d. Samples were run on separate gels and blotted on the same membrane for antibody probing)

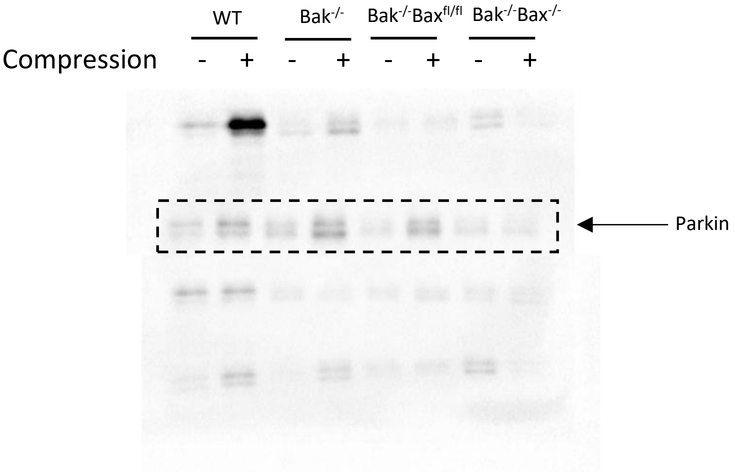

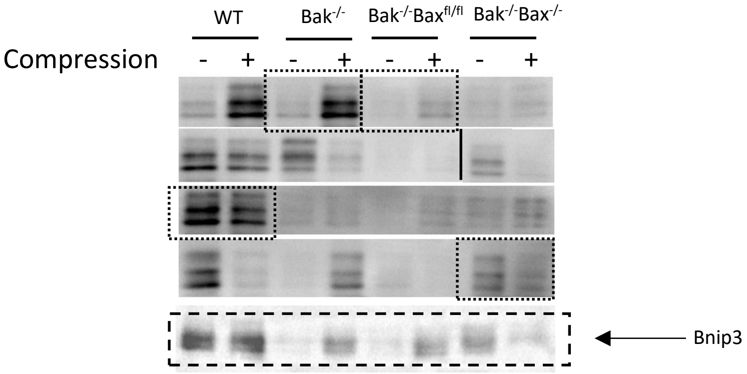

The representative blot of Bnip3 in mitochondria was a re-run of the representative samples after the statistical analysis, which showed the representative analyzed results.

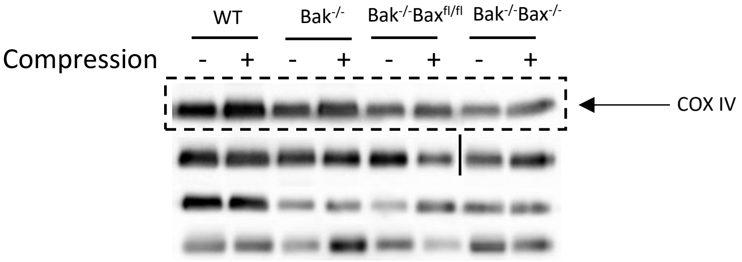

Figure 4e (Raw immunoblots shown in Figure 4e. Samples were run on separate gels and blotted on four membranes for antibody probing. A black solid line is presented to separate two membranes)

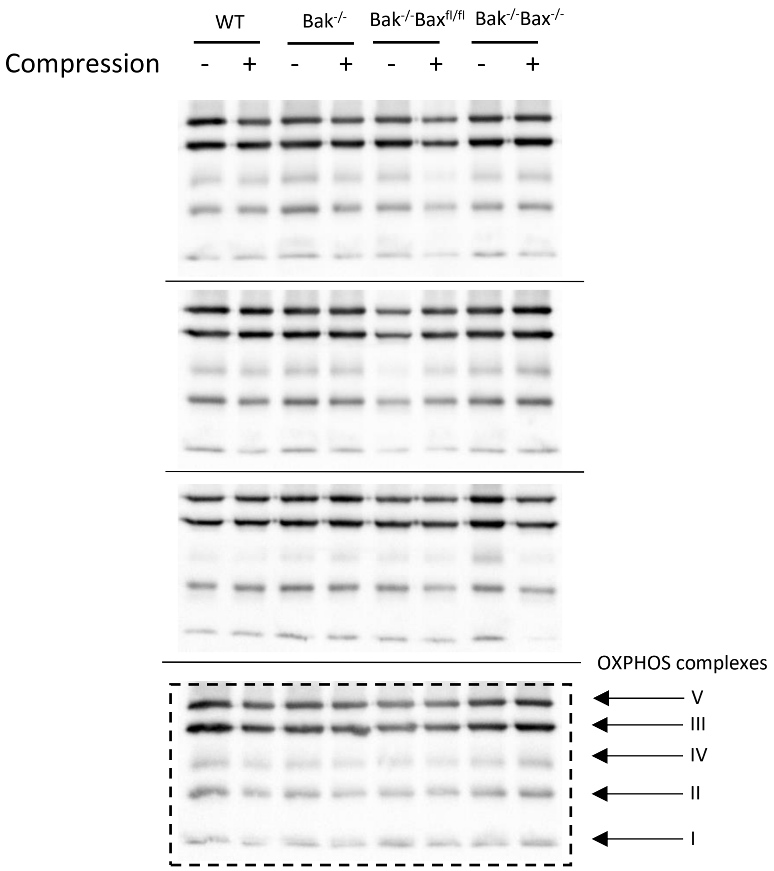

Figure 4f (Raw immunoblots shown in Figure 4f. Samples were run on separate gels and blotted on the same membrane for antibody probing)

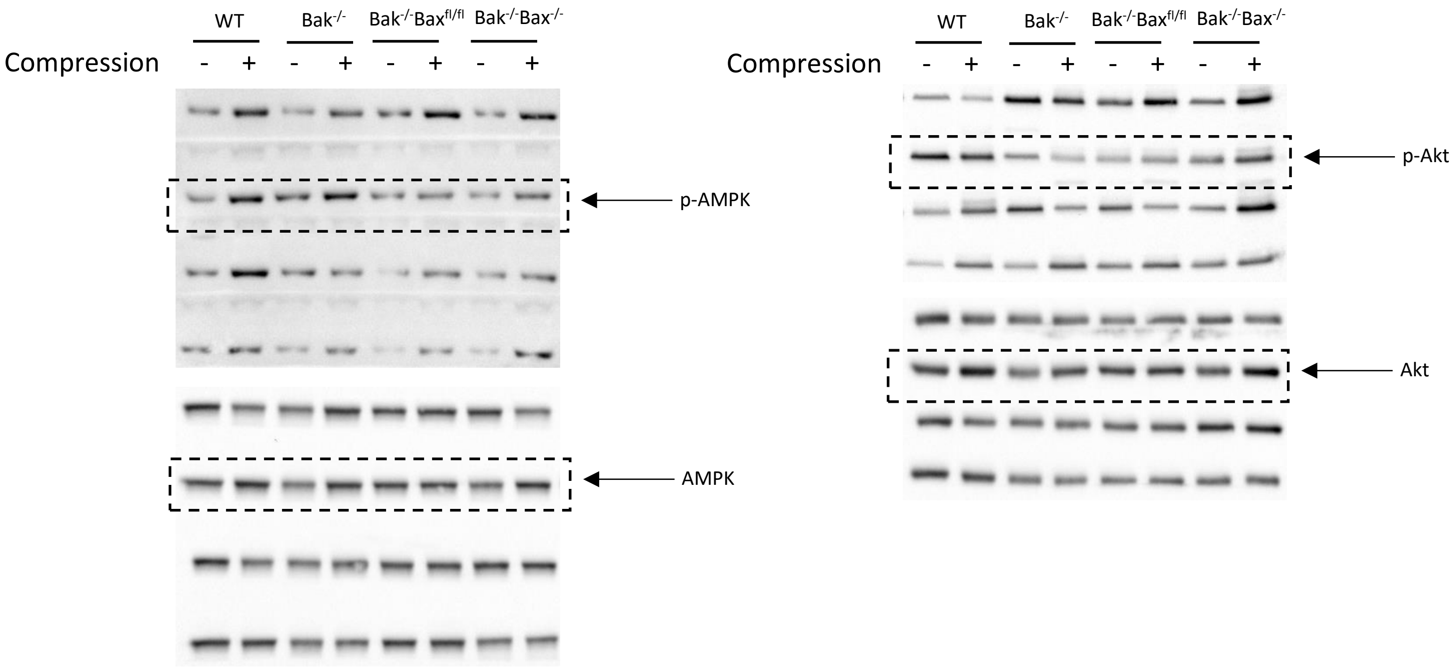

Figure 5a (Raw immunoblots shown in Figure 5a. Samples were run on separate gels and blotted on the same membrane for antibody probing)

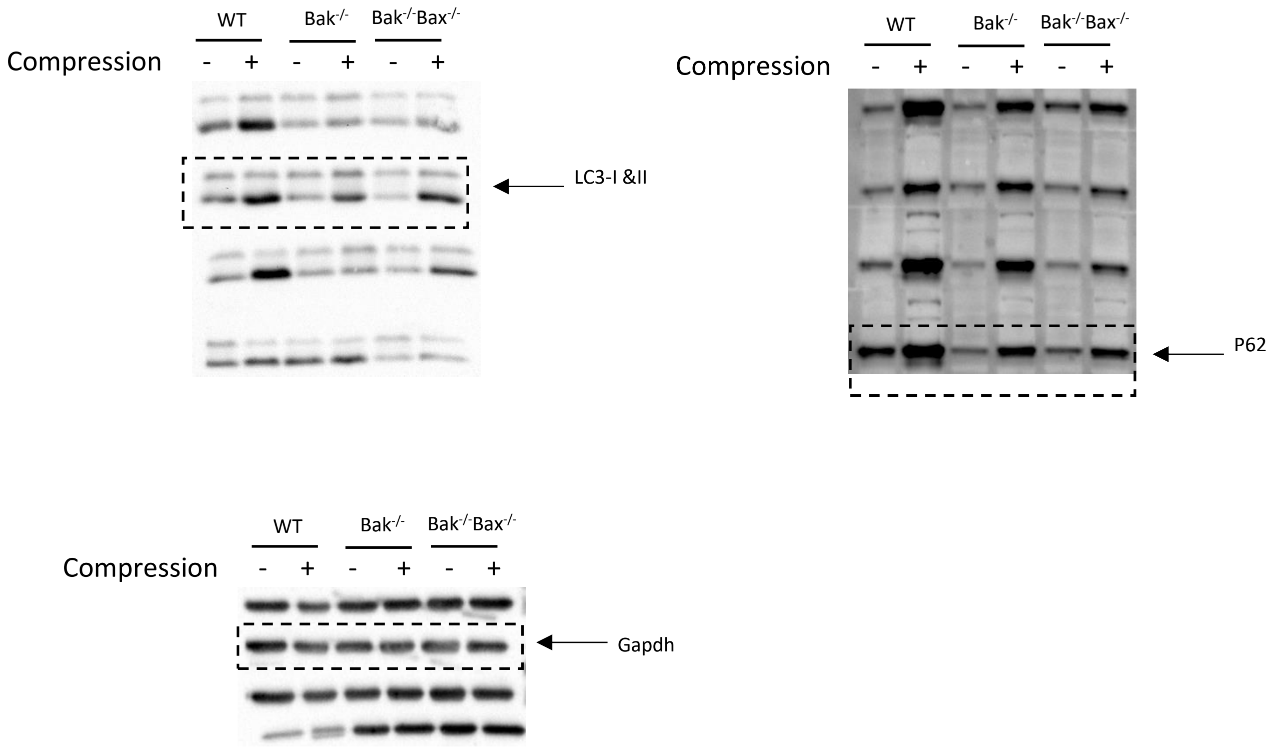

Figure 5b (Raw immunoblots shown in Figure 5b. Samples were run on separate gels and blotted on four membranes for antibody probing. A black solid line is presented to separate two membranes)

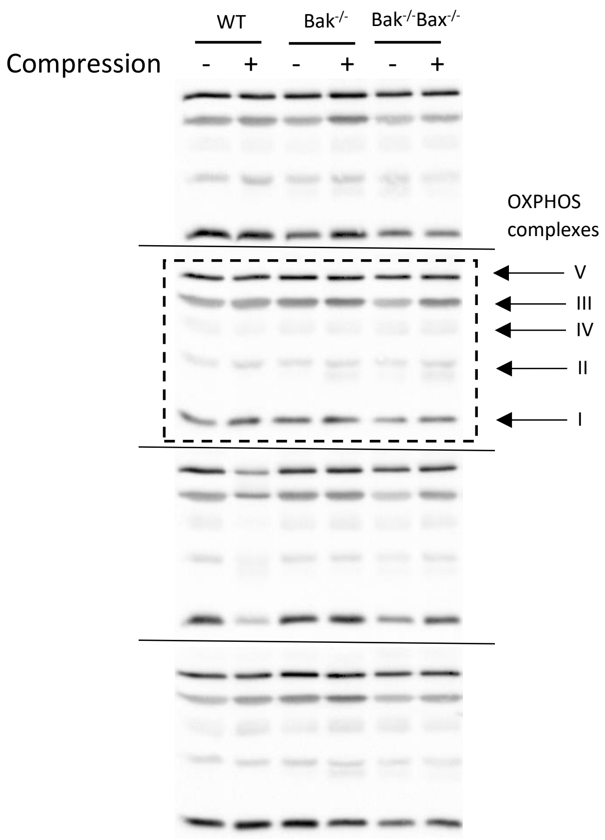

Supplementary Figure 1

a) HSA-MCM

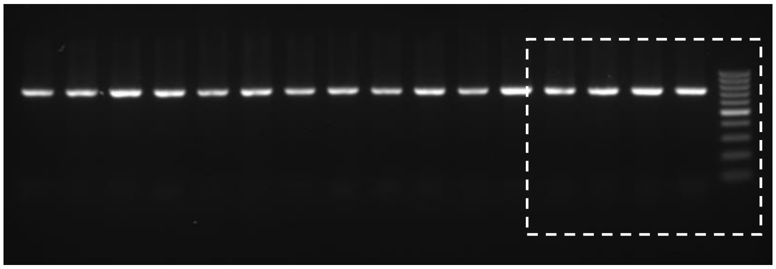

b) Bax$^{fl/fl}$ (Bax1^{tm2Sjk})

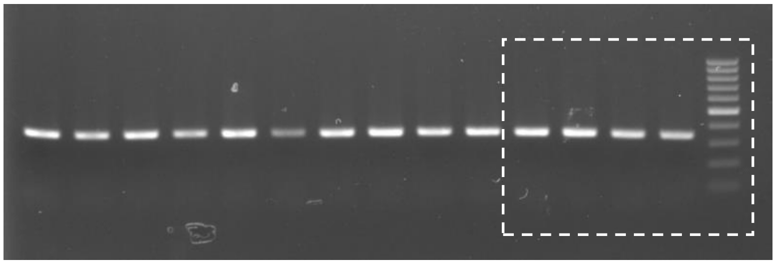

c) Bak$^{-/-}$ (Bak1^{tm1Thsn}) (Samples were run on two gels. A white line is used to separate the two gels)

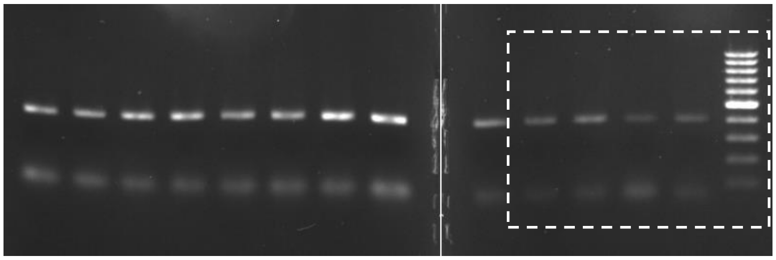

Supplement: Supplementary file 1 — Supplementary Figures [file 41598_2018_21853_MOESM1_ESM.pdf]
